# Performance of the *QPLEX™ Alz plus assay*, a novel multiplex kit for screening cerebral amyloid deposition

**DOI:** 10.1186/s13195-020-00751-x

**Published:** 2021-01-06

**Authors:** Jong-Chan Park, Keum Sim Jung, Jiyeong Kim, Ji Sung Jang, Sunghoon Kwon, Min Soo Byun, Dahyun Yi, Gihwan Byeon, Gijung Jung, Yu Kyeong Kim, Dong Young Lee, Sun-Ho Han, Inhee Mook-Jung

**Affiliations:** 1 Department of Biochemistry and Biomedical Sciences, College of Medicine, Seoul National University, Seoul, 03080 Republic of Korea; 2 Department of Biochemistry & Biomedical Sciences, SNU Dementia Research Center, College of Medicine, Seoul National University, 103 Daehak-ro, Jongno-gu, Seoul, 03080 Republic of Korea; 3 Department of Biochemistry & Biomedical Sciences, Neuroscience Research Institute, Medical Research Center, College of Medicine, Seoul National University, 103 Daehak-ro, Jongno-gu, Seoul, 03080 Republic of Korea; 4 Department of Neurodegenerative Disease, UCL Queen Square Institute of Neurology, University College London, London, WC1E 6BT UK; 5 QuantaMatrix Inc., Seoul, 03080 Republic of Korea; 6 Department of Neuropsychiatry, Seoul National University Bundang Hospital, Seongnam, 13620 Republic of Korea; 7 Department of Neuropsychiatry, Seoul National University Hospital, 101 Daehak-ro, Jongno-gu, Seoul, 03080 Republic of Korea; 8 Department of Nuclear Medicine, SMG-SNU Boramae Medical Center, Seoul, 07061 Republic of Korea; 9 Department of Psychiatry, College of medicine, Seoul National University, Seoul, 03080 Republic of Korea; 10 Institute of Human Behavioral Medicine, Medical Research Center, Seoul National University, Seoul, 03080 Republic of Korea

**Keywords:** Alzheimer’s disease, Pittsburgh compound B, Cerebral amyloid deposition, Blood-based biomarker, *QPLEX™ Alz plus assay*

## Abstract

**Background:**

Alzheimer’s disease (AD) is an irreversible neurodegenerative disease characterized by the hallmark finding of cerebral amyloid deposition. Many researchers have tried to predict the existence of cerebral amyloid deposition by using easily accessible blood plasma samples, but the effectiveness of such strategies remains controversial.

**Methods:**

We developed a new multiplex kit, the *QPLEX™ Alz plus assay kit,* which uses proteomics-based blood biomarkers to prescreen for cerebral amyloid deposition. A total of 300 participants who underwent Pittsburgh compound B (PiB)-positron emission tomography (PET) which allows imaging of cerebral amyloid deposition were included in this study. We compared the levels of *QPLEX™* biomarkers between patients who were classified as PiB-negative or PiB-positive, regardless of their cognitive function. Logistic regression analysis followed by receiver operating characteristic (ROC) curve analysis was performed. The kit accuracy was tested using a randomized sample selection method.

**Results:**

The results obtained using our assay kit reached 89.1% area under curve (AUC) with 80.0% sensitivity and 83.0% specificity. Further validation of the *QPLEX™ Alz plus assay kit* using a randomized sample selection method showed an average accuracy of 81.5%.

**Conclusions:**

Our *QPLEX™ Alz plus assay kit* provides preliminary evidence that it can be used as blood marker to predict cerebral amyloid deposition but independent validation is needed.

**Supplementary Information:**

The online version contains supplementary material available at 10.1186/s13195-020-00751-x.

## Background

Alzheimer’s disease (AD) is a neurological disease accompanied by the pathological features of beta-amyloid (Aβ) plaques and neurofibrillary tangles. AD is the most prevalent dementia and has a much earlier pathological progress than the onset of clinical symptoms; thus, many research efforts have sought to discover bio-fluidic biomarkers in the blood or cerebral spinal fluid (CSF) that can be used for early detection of the disease. Although direct brain-imaging methods using Aβ-specific positron emission tomography (PET) ligands have been developed, such as Pittsburgh compound B (PiB) and florbetapir [1], PET is not an easily accessible method because of its high cost and radiation exposure. Especially in the early stages of the disease, when pathological hallmarks exist in the brain but no clinical symptom is seen, patients would be unlikely to undergo a brain PET scan [2, 3]. This a major obstacle when early diagnosis relies on PET. Many researchers and clinicians have noted that the use of efficient, early, and easily accessible diagnostic methods could prevent or delay the progress of AD pathology.

In a previous study, we revealed a novel blood-based biomarker panel for cerebral amyloid deposition consisting of galectin-3 binding protein (LGALS3BP), Aβ1–40, angiotensin-converting enzyme (ACE), and periostin (POSTN) [3, 4]. When assessed by logistic regression analysis and receiver operating characteristic (ROC) curve analysis, our biomarker panel exhibited a high area under the curve (AUC) and good sensitivity and specificity when blood levels were quantified through commercialized enzyme-linked immunosorbent assay (ELISA) kits or the xMAP technology. For clinical practice and large population screening, a readily accessible diagnostic kit capable of measuring these biomarkers could critically enable the quick prediction of cerebral amyloid deposition.

Here, we introduce a bioanalytical platform for AD diagnosis. We developed a new AD diagnostic kit, the *QPLEX™ Alz plus assay kit,* which predicts cerebral amyloid deposition using our previously developed blood biomarkers, LGALS3BP, Aβ1–40, ACE, and POSTN. In our prior work, these biomarkers were independently quantified using individual ELISA kits. Here, we combined them and developed a new multiplex kit with high efficiency and accuracy. We tested the diagnostic efficacy and accuracy of the kit for 300 cognitively diverse individuals who underwent PiB-PET scans. The new *QPLEX™ Alz plus assay kit* demonstrated high-level performance for prediction of PiB-PET positivity.

## Methods

### Participants

In total, 300 participants were included in this study. They consisted of 149 cognitively normal individuals (CN group), 87 patients with amnestic mild cognitive impairment (MCI group), and 64 patients with clinically diagnosed AD dementia (DEM group). These individuals were recruited as part of the Korean Brain Aging Study for the Early diagnosis and prediction of Alzheimer’s disease (KBASE). All participants were given appropriate clinical and neuropsychological assessments according to the KBASE assessment protocol. The details of participant recruitment, clinical diagnosis criteria, and further information were described in our previous report [5].

### Ethical approval

All participants and (where applicable) their legal representatives read and confirmed the informed consent documents. This project was approved by the Seoul National University Hospital Institutional Review Board.

### PiB-PET

All participants underwent PiB-PET scans using a 3.0 T PET-MR scanner (Siemens Healthineers, Erlangen, Germany). Briefly, each individual was injected intravenously with 555 MBq of [^11^C] PiB (450–610 MBq) PET tracer, which enabled visualization of cerebral amyloid deposition. The degree of amyloid accumulation was calculated by the standardized uptake value ratio (SUVR), which was determined by the automatic anatomic algorithm. The four regions of interest (ROIs) were the lateral temporal, lateral parietal, posterior cingulate-precuneus, and frontal regions. If the SUVR value was 1.4 or higher for at least one of the four ROIs, the individual was defined as PiB-positive (PiB+). The details of the imaging protocols were described in our previous paper [5].

### Blood sampling

All fasting blood samples were collected at 9:00 AM. Whole-blood samples were gathered in K2 EDTA tubes (BD Vacutainer Systems, Plymouth, UK) and centrifuged at 700 g for 5 min at room temperature (RT). The supernatants were collected, the centrifugation step was repeated, and the tubes were stored at − 80 °C.

### *QPLEX™ Alz plus assay* (*QPLEX*™)

Our *QPLEX™* kit utilized the Quantamatrix’s multiplex diagnostics platform (QMAP; Quantamatrix Inc., Seoul, Republic of Korea) with microdisk technology to perform multiplex analyses in a single well [6]. This suspension bead array system uses graphically coded beads to expose antigens to the 3D environment. Briefly, human plasma samples were diluted in the diluent buffer and incubated with the coded beads and biotin-conjugated detection antibodies in the provided black 96-well plate for 90 min at RT on a shaking incubator at 1000 rpm. The immunocomplexes, including the coded beads, were washed twice with washing buffer on a Biotek-510 magnetic wash station (Biotek, VT, USA). Fifty microliters of diluted R-phycoerythrin-conjugated streptavidin were added to each well, and the plate was incubated for 15 min at RT on the same shaking incubator. After three washes, the immunocomplexes were resuspended in 100 μl of washing buffer by tapping. Collected immunocomplexes were analyzed automatically by the QMAP™ system; approximately 30 beads were used for the calculation of each biomarker concentration.

### In-house ELISA (IH-ELISA)

ELISA plates were coated with capture antibodies diluted to a working concentration in Dulbecco’s modified phosphate-buffered saline solution (DPBS; Invitrogen, Carlsbad, CA, USA) and incubated at RT. The remaining binding sites were blocked with 1 mg/ml bovine serum albumin (Sigma Aldrich, St. Louis, MO, USA) in DPBS. Human plasma samples were diluted in the same buffer that was provided with the *QPLEX™* kit and incubated with the primary antibodies immobilized in the wells. Streptavidin-conjugated horseradish peroxidase was added to the wells, and the plate was incubated again for 20 min. The immunocomplexes were detected with a chromogenic substrate solution and the reaction was terminated by the addition of 0.5 M HCl. Absorption was read at 450 nm using an ELISA plate reader (Biotek, Winooski, VT, USA).

### Statistical analyses

All statistical analyses were performed using the Medcalc 17.2 software (Ostend, Belgium) and GraphPad Prism 8 (San Diego, CA, USA). Comparison analyses between two variables were conducted by independent *t* test or analysis of covariance (ANCOVA) with correction for age and sex. Correlation analyses were performed using the Pearson’s correlation analysis method. To calculate the discriminatory power, sensitivity, and specificity for the biomarker panels, logistic regression, followed by receiver operating characteristic (ROC) curve analysis was performed. The basic formula of our algorithm used in our analyses was as follows:

$$ {p}_i=\frac{e^{\left({a}_1\times \mathrm{A}\upbeta 40+{a}_2\times \mathrm{A}\mathrm{CE}+{a}_3\times \mathrm{LGALS}3\mathrm{BP}+{a}_4\times \mathrm{POSTN}+C\right)}}{1+{e}^{\left({a}_1\times \mathrm{A}\upbeta 40+{a}_2\times \mathrm{A}\mathrm{CE}+{a}_3\times \mathrm{LGALS}3\mathrm{BP}+{a}_4\times \mathrm{POSTN}+C\right)}} $$

(*p*_*i*_, predicted probabilities; *a*_*n*_, coefficient values, e.g., *a*_1_ = 0.008 for Aβ40, *a*_2_ = − 0.0066 for ACE, *a*_3_ = − 0.0007 for LGALS3BP, *a*_4_ = 0.1322 for POSTN; *C*, constant, e.g., *C* = 1.2478. Each biomarker level of the samples was multiplied by coefficient values and *p*_*i*_ was calculated).

The formulas, coefficients, and constants could be optimized since there were appropriate outliers and various logistic regression models. Multicollinearities were checked using the values of variance inflation factors (VIF). Finally, the accuracy of each model was calculated using a randomized sample selection method. Randomizing analysis was used to create random groups with even distributions of age (the variable for case identification was ‘age’). In other words, each group had almost the same average age (group 1, average 71.8, range 56 to 86; group 2, 72.3, range 61 to 84; group 3, 70.1, range 55 to 85; etc.). After the randomized sample selection and regrouping, each group was validated by the logistic regression models to calculate the accuracy of each biomarker panel. The average accuracy was calculated as shown in Fig. 3. All statistical outliers were excluded from the cohort according to the Grubb’s double-side outlier test (*p* <  0.05).

## Results

### Categorization of participants

This study included 300 participants (age, 55–88 years): 113 PiB− CN (CN−), 36 PiB+ CN (CN+), 31 PiB− MCI (MCI−), 56 PiB+ MCI (MCI+), 6 PiB− DEM (DEM−), and 58 PiB+ DEM (DEM+). The demographic details are described in Table 1.

**Table 1:** Demographic data of the participants (*N* = 300)

Characteristics (*n*)	CN− (113)	CN+ (36)	MCI− (31)	MCI+ (56)	DEM− (6)	DEM+ (58)	*P* value
Gender, M/F	46/67	20/16	10/21	17/39	2/4	18/40	> 0.1^†^
Age, years, mean ± SEM	67.32 ± 0.8	74.47 ± 1.0	73.58 ± 0.1	73.23 ± 0.9	75.66 ± 4.3	72.28 ± 1.0	< 0.001*
Education, mean ± SEM	11.39 ± 0.5	12.08 ± 0.8	9.2 ± 0.8	10.16 ± 0.6	6.00 ± 1.7	9.67 ± 0.7	< 0.01*
MMSE, mean ± SEM	26.96 ± 0.2	27.16 ± 0.4	22.93 ± 0.6	21.73 ± 0.4	18.17 ± 3.0	16.59 ± 0.5	< 0.001*
MMSE *z*, mean ± SEM	0.29 ± 0.1	0.44 ± 0.1	− 0.91 ± 0.2	− 1.54 ± 0.2	− 2.08 ± 0.2	− 3.23 ± 0.2	< 0.001*
CDR (*n*)	0 (113)	0 (36)	0.5 (31)	0.5 (56)	0.5 (2), 1 (4)	0.5 (17), 1 (41)	< 0.001^†^
ApoE4 status, ε4+/*N*	20/113	12/36	2/31	33/56	0/6	40/58	< 0.001^†^
PiB (SUVR), mean ± SEM	1.10 ± 0.01	1.62 ± 0.05	1.13 ± 0.01	1.92 ± 0.04	1.10 ± 0.02	2.10 ± 0.05	< 0.001*

*CN* cognitively normal, *MCI* mild cognitive impairment, *DEM* clinically dementia, *PiB* Pittsburgh compound B, − or +, PiB positivity, *SEM* standard error of mean, *n* number of participants, *MMSE* Mini-Mental State Examination, *MMSE z score* a revised value of the MMSE score with consideration for age, gender, and education level, *CDR* Clinical Dementia Rating, *ApoE* Apolipoprotein E, *SUVR* standardized uptake value ratio, *N* total number of participants of each group

*Significance by one-way analysis of variance test (ANOVA)

^†^Significance by chi-squared test

### Comparison between IH-ELISA and *QPLEX*™ kit performance

**Fig. 1:**
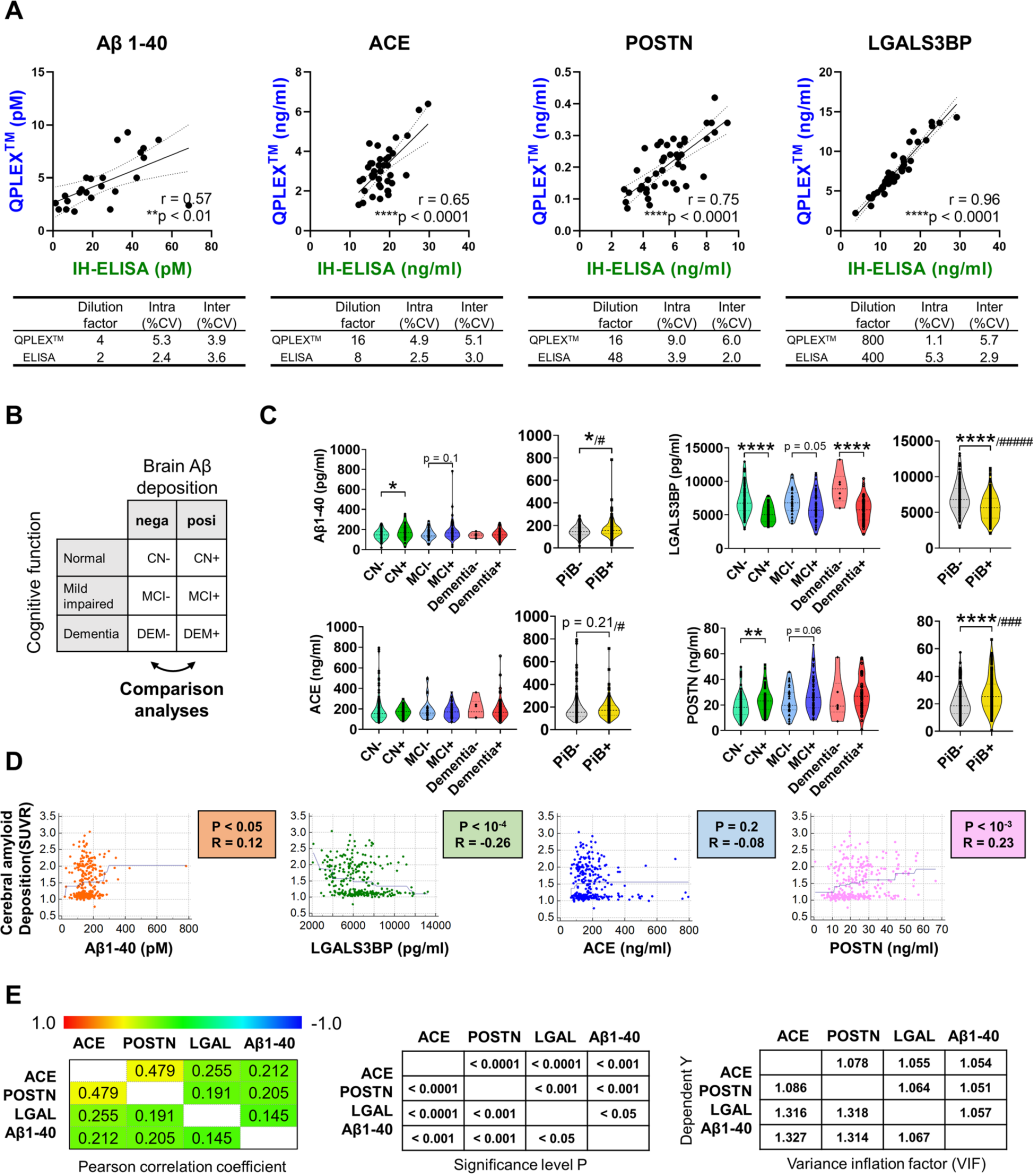
The levels of *QPLEX™* biomarkers and their correlation to cerebral amyloid deposition. **a** Correlation between IH-ELISA and *QPLEX™* biomarkers and inter- or intra-CV. Significant outliers were excluded by the Grubb’s test method. **b** Classification of participants. Participants were divided into two groups (cerebral amyloid deposition; − or + means PiB− vs. PiB+) or three groups (depending on their cognitive status; CN normal, MCI mild cognitive impaired, Dementia). Comparison analyses for PiB− vs. PiB+ were conducted. **c** The levels of *QPLEX™* biomarkers. *p <  0.05, **p <  0.01, independent *t* test; ^#^*p* <  0.10, ^###^*p* <  0.001, ^#####^*p* <  0.0001, ANCOVA test with the correction of age, sex, ApoE, and/or MMSE score. **d** Correlation between cerebral amyloid deposition and *QPLEX™* biomarkers. **e** Inter-correlations between the markers (Pearson’s correlation coefficient, *p* values, and variance inflation factor)

We previously reported a novel blood-based biomarker panel identified from the results of a proteomic analysis [4]. In the prior work, we used four different commercially available ELISA kits to assess the individual biomarkers. Here, we developed a new multiplex strategy. Before seeking to validate our *QPLEX™* kit using the main cohort samples, we compared the results obtained for these markers between IH-ELISA and our kit. All four biomarkers included in the kit showed a high correlation coefficient and low intra- or inter-coefficient of variation (CV, < 10%) (Fig. 1a). This indicated that our *QPLEX™* kit could show high performance similar to that achieved with IH-ELISA of the individual biomarkers.

### Validation of *QPLEX*™ biomarkers for predicting PiB-PET positivity

We divided all participants into two groups, PiB− (CN−, MCI−, and DEM−) and PiB+ (CN+, MCI+, DEM+), and compared the levels of *QPLEX™* biomarkers (Fig. 1b). Similar to the results reported in our previous paper [4], all *QPLEX™* biomarkers showed significant differences between the PiB− and PiB+ groups (Fig. 1c). There were several intra-subgroup differences (parentheses indicate markers that showed significant between-subgroup differences): CN− vs. CN+ (Aβ1–40, LGALS3BP, and POSTN), MCI− vs. MCI+ (Aβ1–40, LGALS3BP, and POSTN) and DEM− vs. DEM+ (LGALS3BP) (Fig. 1c). Moreover, the *QPLEX™* biomarkers exhibited correlations with the degree of cerebral amyloid deposition (PiB-PET standardized uptake value ratio; PiB-PET SUVR) with the exception of ACE, which exhibited a non-significant tendency (*p* = 0.2) (Fig. 1d). ACE did, however, show a significant association with PiB-PET SUVR in the multiple regression analysis (Table 2). Finally, we found that all of the *QPLEX™* biomarkers were associated with each other (Fig. 1e, left and middle). The low variance inflation factors (VIF < 2) revealed that there was no problem with multicollinearity (Fig. 1e, right). Together, these results confirmed that our *QPLEX™* biomarkers appear appropriate for use in prescreening patients for cerebral amyloid deposition.

**Table 2:** Multiple regression analysis

QPLEX™ markers							
Dependent Y		**Cerebral amyloid deposition (SUVR)**					
Sample size						292	
Coefficient of determination *R*^2^						0.1925	
*R*^2^-adjusted						0.1813	
Multiple correlation coefficient						0.4388	
Residual standard deviation						0.4401	
Ind. variables	**Coefficient**	**Std. error**	***t***	***P***	***r***_**partial**_	***r***_**semipartial**_	**VIF**
(Constant)	1.6344						
Aβ1–40	0.0009742	0.0004063	2.397	0.0171	0.1401	0.1272	1.065
LGALS3BP	− 0.00007365	0.00001310	− 5.623	< 0.0001	− 0.3150	0.2983	1.075
ACE	− 0.0008671	0.0002836	− 3.057	0.0024	− 0.1776	0.1621	1.342
POSTN	0.01510	0.002555	5.908	< 0.0001	0.3293	0.3134	1.332

*SUVR* standardized uptake value ratio, *Ind*. independent, *VIF* variance inflation factor, *LGALS3BP* galectin-3 binding protein, *ACE* angiotensin-converting enzyme, *POSTN* periostin; ApoE, apolipoprotein E

### Discrimination power of QPLEX™ biomarkers

**Fig. 2:**
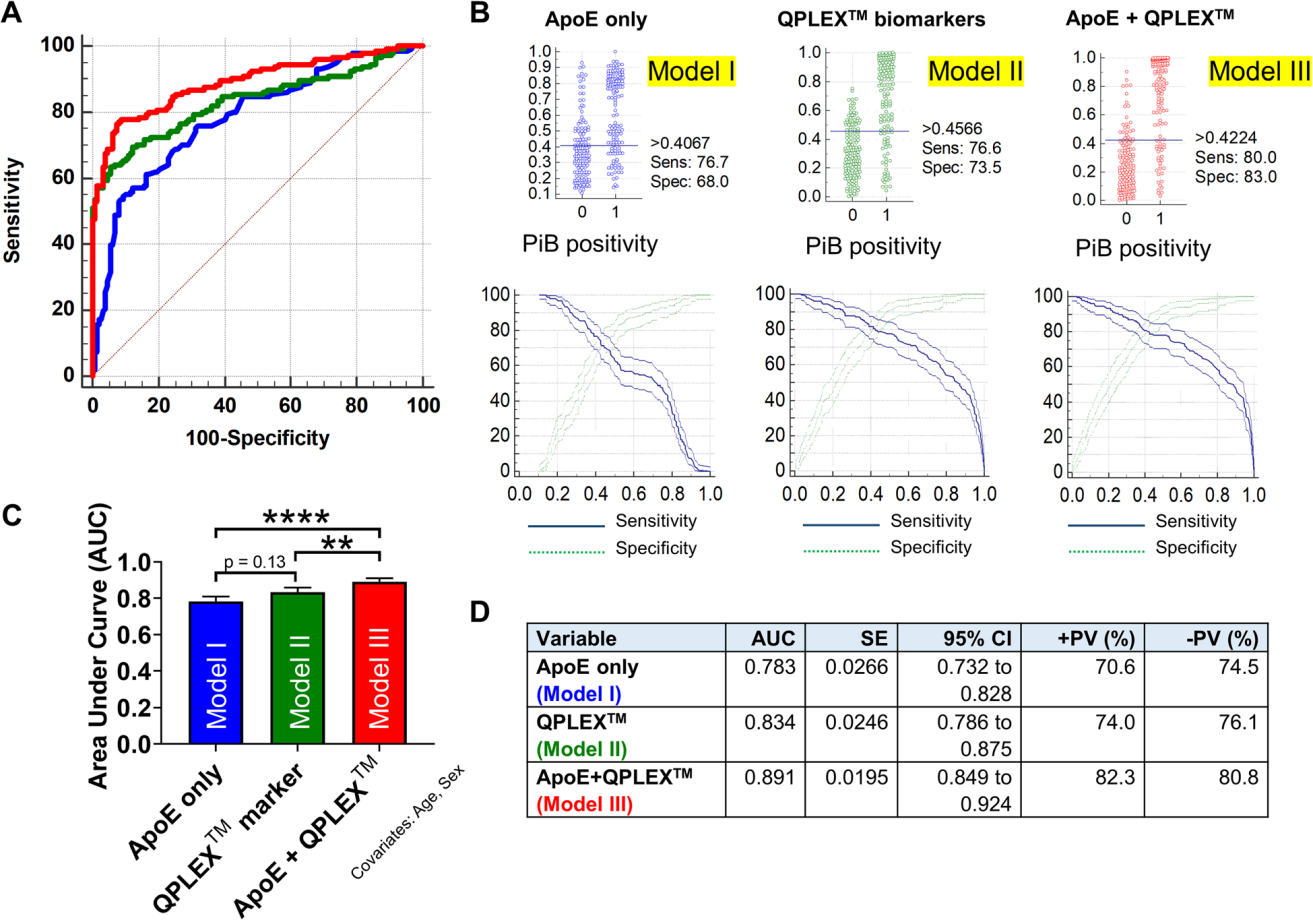
Receiver operating characteristic (ROC) curve analysis for PiB positivity. **a** ROC curve analyses for PiB positivity. Age and sex were used as covariates. **b** Interactive dot diagram and plot versus criterion values showing sensitivity, specificity, and Youden index cutoff criteria. **c**, **d** Comparison of ROC curve analyses. Age sex were used as covariates. Model III had the highest area under curve (AUC) value (0.891). PV, predictive values; SE, standard error; CI, confidence interval

To verify the usefulness of the *QPLEX™* biomarkers, we performed logistic regression analysis and receiver operating characteristics (ROC) curve analysis (Fig. 2a, Table 3). We generated three regression models (ApoE variable only, model I; *QPLEX™* markers only, model II; ApoE + *QPLEX™* markers, model III) with correction for age and sex (Fig. 2b). Model II showed a higher AUC (0.834 with 76.6% sensitivity and 73.5% specificity) than model I (0.783 with 76.7% sensitivity and 68.0% specificity), and model III showed a significantly higher AUC (0.891 with 80.0% sensitivity and 83.0% specificity) than models I and II (Fig. 2c, d). Furthermore, we performed the same analyses for CN or MCI group separately, it also showed the consistent pattern of high performances (for CN group, 0.786 AUC for model I, 0.896 AUC for model II, 0.907 AUC for model III; for MCI group, 0.781 AUC for model I, 0.791 AUC for model II, 0.900 AUC for model III) and similar tendency of AUC with Fig. 2c (model I < model II < model III) (Supplementary Fig. 1). The results indicated that the model combining our *QPLEX™* biomarkers and ApoE genotypic characteristics yielded a significantly higher AUC value than that generated using only ApoE characteristics.

**Table 3:** Details for logistic regression analyses and criterion values from the ROC curves

	Logistic regression				Criterion values		
Model	***n***	Significance	***R***^**2**^	Chi^**2**^	Cutoff	Sensitivity, % (95% CI)	Specificity, % (95% CI)
ApoE	300	< 0.0001	0.32	81.9	> 0.4067	76.67 (69.1–83.2)	68.00 (59.9–75.4)
QPLEX™	292	< 0.0001	0.45	120.8	> 0.4566	76.55 (68.8–83.2)	73.47 (65.6–80.4)
ApoE + QPLEX™	292	< 0.0001	0.59	172.5	> 0.4224	80.00 (72.6–86.2)	82.99 (75.9–88.7)

Eight samples were excluded as outliers by Grubb’s test (*p* < 0.05, double sides)

Age and sex were used as covariates

*R*^*2*^ Nagelkerke *R*^2^, *Chi*^*2*^ chi-squared test, *CI* confidence interval, *ROC* receiver operating characteristics

### Accuracy test using randomized sample selection

**Fig. 3:**
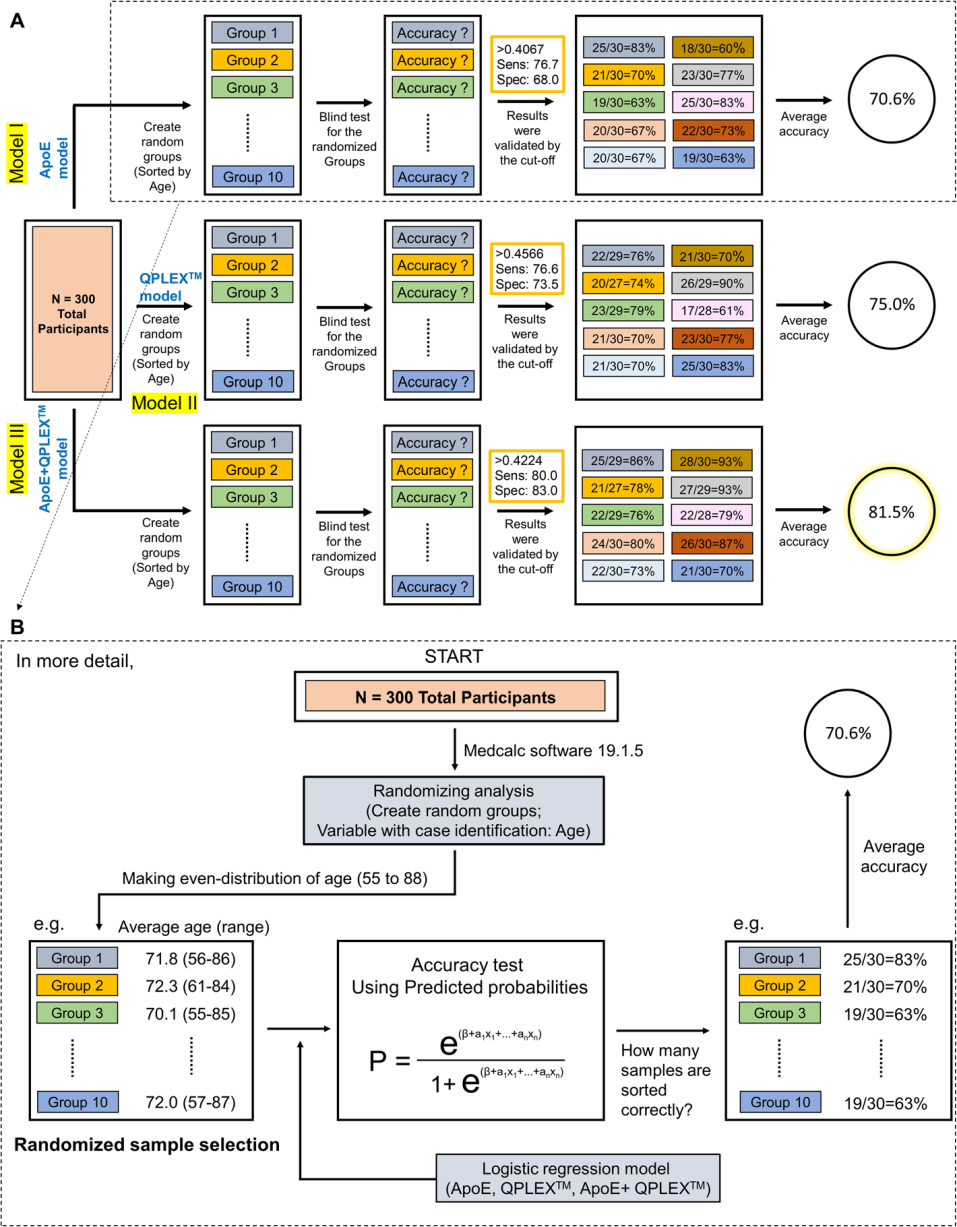
Accuracy validation of *QPLEX™* kit by using randomized sample selection. **a** Random groups were generated (sorted by age) and 10 groups were blind-tested according to our *QPLEX™* algorithm. Three models were tested: model I, 70.6% accuracy; model II, 75.0% accuracy; model III, 81.5% accuracy. **b** The detailed algorithm process to validate our biomarker models by using randomized sample selection. Random groups were generated by randomizing analysis (create random groups using “age” with case identification). Three hundred participants were classified as 10 groups and validated using their predicted probabilities (P). Accuracy (correct number of participants/total number of participants, %) of each group were calculated and averaged

Next, we tested the accuracy of our regression models by using randomized sample validation (Fig. 3a). First, we randomly divided the participants into 10 groups with even distributions of ages 55 to 88 (variable for case identification = “age”; for more detail on the randomized sample selection process, see Fig. 3b). We confirmed that the average ages were almost the same (data not shown). We then tested how many samples could be predicted accurately by models I, II, and III, in each group (e.g., for model I, correct predictions were obtained for 25 out of 30, 83%, for group 1; 21 out of 30, 70%, for group 2; 19 out of 30, 63%, for group 3;… 19 out of 30, 63%, for group 10). Finally, we calculated average accuracies for models I, II, and III. Consistent with the results for the whole-population ROC curve analysis (Fig. 2c, d), model III showed the highest average accuracy (81.5%) compared to model I (70.6%) and model II (75.0%). The results obtained from our *QPLEX™* prediction models suggest that this new method can effectively discriminate PiB-PET-positive and thus is ready to be put to practical use for the general public.

## Discussion

Blood-based biomarkers for AD theoretically should enable early-stage disease detection or screening while also offering increased accessibility, greater convenience, and reduced cost [7]. Various blood biomarkers for AD diagnosis have been published in the last few decades [7–10], but few have proven useful for the development of diagnostic kits. Indeed, no blood-based biomarker kit for diagnosing AD has yet been launched in the market. This likely reflects the technical difficulty of composing a multiplex kit with detection accuracies that are consistently equivalent to those of the individual ELISA experiments used during the discovery and validation stages of a given biomarker.

ELISA has long been used for biomarker detection in diverse body fluids, including blood and CSF [11, 12]; however, it requires intensive labor and typically shows intra- and inter-assay variability [13]. The newly developed xMAP technology exhibits high sensitivity and low variability thanks to its use of pre-made calibrators [14]. Comparative analysis showed that the detection values of ELISA and xMAP were generally (but not always) correlated, the results differed in terms of the absolute concentrations identified [15–17]. Comparison of the CSF Aβ42, t-tau, and p-tau181 levels detected by ELISA and xMAP [14, 18] showed that the levels were well correlated, supporting the idea that multiplex xMAP is an appropriate and potentially clinically relevant platform for biomarker detection [14].

Quantamatrix’s multiplexed diagnostics platform (QMAP™) uses a proprietary microdisk technology to perform 1000 reactions in a single well. The microdisk is formed of a polymer that comprises a large amount of 50-μm particles applied by a semiconductor process, and can easily be formed into various shapes [6]. The microdisk is further coated with silica to improve its physical and chemical stability, and to facilitate various chemical surface treatments [19]. This microdisk-based multiplex technology enhanced the detection ability of our *QPLEX™ Alz plus assay kit* for rare or volume-limited samples. Moreover, the graphical code used by our group is more stable than the fluorescence code which can be affected by light and heat. This high-level stability and the magnetic character make the QMAP™ system suitable for application in automated analysis equipment. The QMAP™-based kit measures blood biomarkers by sandwich ELISA using capture antibodies and biotinylated detection antibodies. The system can perform up to 1000 reactions in a single well with a suspension bead array system that enables 3D incubation and provides fast results within 2 h (excluding sample preparation).

Our *QPLEX™ Alz plus assay kit* was developed using QMAP™ technology and four blood biomarkers we previously identified as being useful for distinguishing cerebral amyloid deposition [3, 4]: LGALS3BP, ACE, POSTN, and Aβ1–40. We validated the diagnostic accuracy of this diagnostic kit in a clinical trial with a 300-person validation cohort of individuals who were imaged using PiB-PET. In the previous discovery stage, we utilized ELISA to detect LGALS3BP, ACE, and POSTN, and used xMAP to detect Aβ1–40; with this strategy, we used the developed kit to validate that there were differences in the blood levels of the four biomarkers between the PiB− and PiB+ groups [3, 4]. In the *QPLEX™ Alz plus assay kit*, we simultaneously detected LGALS3BP, ACE, POSTN, and Aβ1–40 using QMAP technology, which helped maximize the detection efficiency for low-abundance blood proteins.

When assessed with the new assay kit, the blood levels of LGALS3BP, POSTN, and Aβ1–40 showed fairly distinct and significant differences between the PiB− and PiB+ groups and significant correlations with cerebral amyloid deposition (SUVR); the level of ACE tended to be decreased and was correlated with cerebral amyloid deposition in the PiB+ group (Fig. 2b, c). Notably, previous studies showed that aspects of ACE other than the blood concentration appear to be tightly related to AD pathology. For example, decreased ACE activity was found in CSF from AD patients [20] and the ACE D allele was associated with an increased risk for AD in two distinct population cohorts [21]. This increased risk for AD was independent of ApoE4 genotype and was equal to or greater in magnitude than the ApoE4 genotype-related risk of AD [21]. Here, we found that the blood ACE level was also significantly correlated with the levels of two other AD-related blood biomarkers: LGALS3BP and Aβ1–40 (Fig. 2d). Given this, we propose that ACE is associated with AD pathology and that the blood concentration and activity level of ACE should be investigated further in a larger population cohort. We screened the medication history of all participants and confirmed that there was no current use of an ACE-related medication, such as an ACE inhibitor. As ACE inhibitors are known to influence the activity or concentration of ACE in the blood [22], future studies are warranted to examine the possible effects associated with taking ACE inhibitors.

The ApoE genotype currently stands as the strongest known risk factor for late-onset AD [23], but this genetic risk factor does not reflect the risk an aging individual acquires related to the accumulation of life pattern- and environment-related factors. The biomarkers assessed in the *QPLEX™ Alz plus assay kit* represent a real-time risk assessment for the initiation and progression of AD pathology. Therefore, the combined screening of ApoE and blood biomarkers can be used to improve disease diagnosis. Indeed, the results of our ROC curve analysis revealed that the AUC value of the *QPLEX™ Alz plus assay kit* was higher than that of ApoE alone (0.834 and 0.783, respectively) and that their use in combination significantly increased the AUC value (up to 0.891) compared to that obtained with either strategy alone (Fig. 3). Furthermore, the diagnostic accuracy of our developed kit is comparable or superior to those from other tests using well-known plasma AD biomarkers such as plasma Aβ42/40 ratio, Aβ40/42 ratio, or plasma phosphorylated-tau (p-tau) (Supplementary Table 1) [3, 24–32]. First, the AUC value from Model III (0.891 with 80.0% sensitivity and 83.0% specificity; the model with ApoE covariate) was the highest rank among the models using ApoE as a covariate (1st out of 3 biomarkers). Next, when we compared our AUC value from model II (0.834 with 76.6% sensitivity and 73.5% specificity; the model without ApoE covariate) to those of the tests using these known biomarkers without ApoE covariate, it was also comparable to them (4th out of 10 biomarkers). Our result is fairly promising in that this kit could be easily commercialized and utilized with high accuracy, considering the following limitations of the four tests with higher AUC than that of us: (i) immunoprecipitation-mass spectrometry (IP-MS) method is not accessible to most clinical laboratories [31], (ii) Perez-Grijalba’s approach has a limitation in that the number of participants is too small (*n* = 59) [29], and (iii) it is still necessary to discuss practicality of p-tau, since its level in the blood is too low [24]. Of course, our model also needs to be replicated in an independent cohort, but instead, the results from ROC curve analysis were supported by another validation study in which we used randomized subgroups (see Fig. 3). We found that the accuracy of the *QPLEX™ Alz plus assay kit* (75.0%) was better than that of the ApoE genotype (70.6%), and the highest accuracy was achieved using both together (81.5%). The results provide preliminary evidence that this new assay kit can be used as a blood biomarker panel to predict cerebral amyloid deposition if further validation using an independent cohort is completed.

### Limitations

Although the efficacy of our assay kit was further validated by the randomized selection methods (Fig. 3), our new *QPLEX Alz plus assay kit* should be screened on other independent cohorts with the goal of putting it to practical use. Going forward, we plan to apply the *QPLEX Alz plus assay kit* to a larger patient population and potentially seek to identify a better algorithm. Also, we need to test the *QPLEX Alz plus assay kit* in other neurodegenerative diseases in our future study to validate the selective effects of discriminating PiB-PET-positivity regardless of disease types.

## Conclusions

Our *QPLEX™ Alz plus assay kit* shows high performance and provides the possibility of clinical application for prediction of cerebral amyloid deposition but independent validation is needed.

## Supplementary Information

**Additional file 1: Supplementary Fig. 1.** Comparison of PiB^−^ vs. PiB^+^ among CN or MCI. (A) Logistic regression analysis followed by ROC curve analysis among the CN group and comparison of ROC curve analysis. (B) Logistic regression analysis followed by ROC curve analysis among the MCI group and comparison of ROC curve analysis. AUC, area under curve; PV, predictive values; SE, standard error; CI, confidence interval.

**Additional file 2: Supplementary Table 1.** Recent studies (< 5 years) published on data using plasma p-tau or Aβ42/40 or 40/42 ratio biomarkers, for brain Aβ positivity.

## Data Availability

Not applicable.

## Abbreviations

AD: Alzheimer’s disease
Aβ: Beta-amyloid
CSF: Cerebral spinal fluid
PET: Positron emission tomography
PiB: Pittsburgh compound B
LGALS3BP: Galectin-3 binding protein
ACE: Angiotensin-converting enzyme
POSTN: Periostin
ROC: Receiver operating characteristics
AUC: Area under curve
PV: Predictive value
ELISA: Enzyme-linked immunosorbent assay
CN: Cognitively normal
MCI: Mild cognitive impairment
DEM: Clinically diagnosed AD dementia
KBASE: Korean Brain Aging Study for the Early diagnosis and prediction of Alzheimer’s disease
SUVR: Standardized uptake value ratio
ROI: Region of interest
QMAP: Quantamatrix’s multiplex diagnostics platform
ANCOVA: Analysis of covariance
VIF: Variance inflation factors
ApoE: Apolipoprotein E
