# Supplementary figures and images for: Performance of the QPLEX™ Alz plus assay, a novel multiplex kit for screening cerebral amyloid deposition
Source: Alzheimers Res Ther. 2021 Jan 6;13:12. doi: 10.1186/s13195-020-00751-x (PMC7786945; doi:10.1186/s13195-020-00751-x)

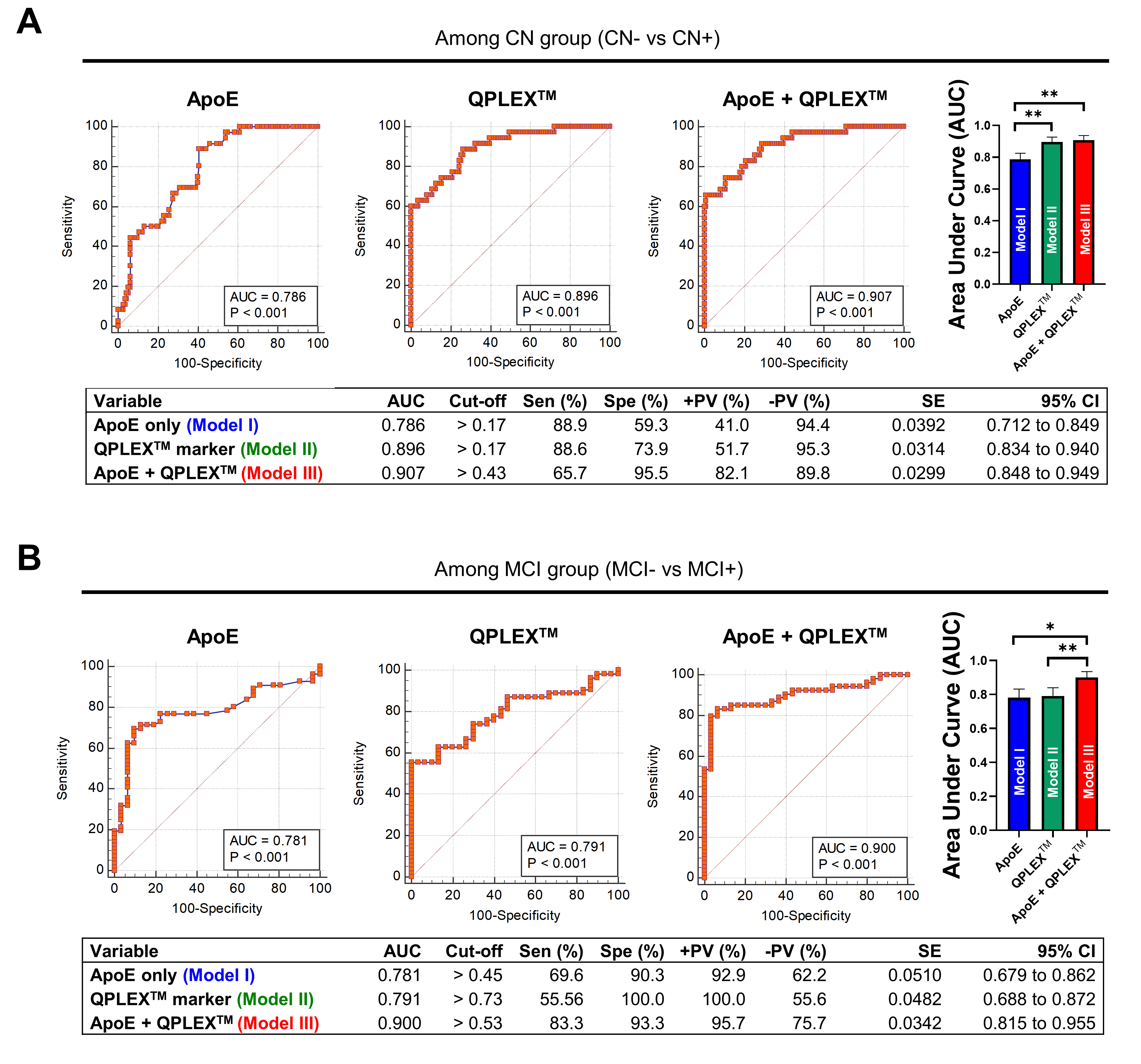

Supplement: Supplementary file 1 — Additional file 1: Supplementary Fig. 1. Comparison of PiB− vs. PiB+ among CN or MCI. (A) Logistic regression analysis followed by ROC curve analysis among the CN group and comparison of ROC curve analysis. (B) Logistic regression analysis followed by ROC curve analysis among the MCI group and comparison of ROC curve analysis. AUC, area under curve; PV, predictive values; SE, standard error; CI, confidence interval. [file 13195_2020_751_MOESM1_ESM.tif]
