# Supplementary material for: Performance of the QPLEX™ Alz plus assay, a novel multiplex kit for screening cerebral amyloid deposition
Source: Alzheimers Res Ther. 2021 Jan 6;13:12. doi: 10.1186/s13195-020-00751-x (PMC7786945; doi:10.1186/s13195-020-00751-x)
Supplement: Supplementary file 2 — Additional file 2: Supplementary Table 1. Recent studies (< 5 years) published on data using plasma p-tau or Aβ42/40 or 40/42 ratio biomarkers, for brain Aβ positivity. [file 13195_2020_751_MOESM2_ESM.docx]

**Supplementary Table 1. Recent studies (< 5 years) published on data using plasma p-tau or Aβ42/40 or 40/42 ratio biomarkers, for brain Aβ positivity.**

| Biomarker | N (PET-/+) | AUC | 95% CI | Sensitivity (%) | Specificity (%) | Plasma quantification method  /Reference standard for brain beta-amyloid status | Covariates | PMID (Reference number) |
| --- | --- | --- | --- | --- | --- | --- | --- | --- |
| Plasma p-tau (Thr 181) | 95/90 | 0.914 | 0.869-0.958 | 88.9 | 85.3 | Meso Scale Discovery  /PiB-PET | age | 32123386 (1) |
|  | 145/81 | 0.848 | 0.802-0.893 | n/a | n/a | Simoa technology  /^18^F-AZD4694-PET | age, Apoe | 32333900 (2) |
|  | 131/138 | 0.803 | 0.749-0.856 | n/a | n/a | Meso Scale Discovery  /PiB-PET | none | 29626426 (3) |
| Plasma Aβ 42/40 ratio | 253/100 | 0.799 | 0.746-0.852 | 78.0 | 66.8 | Bioplex200  /PiB-PET | age, sex, Apoe | 28330509 (4) |
|  | 50/50 | 0.580 | 0.460-0.700 | 61.2 | 63.6 | ABtest40 and ABtest42 by Araclon Biotech Ltd  /Clinical diagnosis | none | 32176645 (5) |
|  | 16/28 | 0.775 | n/a | n/a | n/a | ABtest40 and ABtest42 by Araclon Biotech Ltd  /^18^F-Florbetapir-PET | age, sex | 31384662 (6) |
|  | 67/68 | 0.768 | 0.686-0.849 | 56.7 | 92.6 | Neurology 3-plex A kit  /PiB-PET | age | 32123386 (1) |
|  | 474/368 | 0.770 | 0.740-0.810 | 75.0 | 72.0 | Elecsys immunoassays on a cobas e 601 analyzer (Roche Diagnostics)  /CSF-based standard | none | 31233127 (7) |
|  | 41/18 | 0.881 | 0.779-0.982 | 77.8 | 87.5 | ABtest40 and ABtest42 by Araclon Biotech Ltd  /PiB-PET | none | 31787105 (8) |
| Plasma Aβ 40/42 ratio | 203/73 | 0.794 | n/a | 78.1 | 74.9 | Simoa technology  /^18^F-Florbetapir-PET | none | 31113759 (9) |
|  | 122/110 | 0.935 | 0.904-0.965 | 92.7 | 80.3 | IP-mass spectrometry  /PiB-PET | none | 29420472 (10) |

*n/a: non-available from the papers

**References for Supplementary Table 1.**

1. Thijssen EH, La Joie R, Wolf A, Strom A, Wang P, Iaccarino L, et al. Diagnostic value of plasma phosphorylated tau181 in Alzheimer's disease and frontotemporal lobar degeneration. Nat Med. 2020;26(3):387-97.

2. Karikari TK, Pascoal TA, Ashton NJ, Janelidze S, Benedet AL, Rodriguez JL, et al. Blood phosphorylated tau 181 as a biomarker for Alzheimer's disease: a diagnostic performance and prediction modelling study using data from four prospective cohorts. Lancet Neurol. 2020;19(5):422-33.

3. Mielke MM, Hagen CE, Xu J, Chai X, Vemuri P, Lowe VJ, et al. Plasma phospho-tau181 increases with Alzheimer's disease clinical severity and is associated with tau- and amyloid-positron emission tomography. Alzheimers Dement. 2018;14(8):989-97.

4. Park JC, Han SH, Cho HJ, Byun MS, Yi D, Choe YM, et al. Chemically treated plasma Abeta is a potential blood-based biomarker for screening cerebral amyloid deposition. Alzheimers Res Ther. 2017;9(1):20.

5. Feinkohl I, Schipke CG, Kruppa J, Menne F, Winterer G, Pischon T, et al. Plasma Amyloid Concentration in Alzheimer's Disease: Performance of a High-Throughput Amyloid Assay in Distinguishing Alzheimer's Disease Cases from Controls. J Alzheimers Dis. 2020;74(4):1285-94.

6. Risacher SL, Fandos N, Romero J, Sherriff I, Pesini P, Saykin AJ, et al. Plasma amyloid beta levels are associated with cerebral amyloid and tau deposition. Alzheimers Dement (Amst). 2019;11:510-9.

7. Palmqvist S, Janelidze S, Stomrud E, Zetterberg H, Karl J, Zink K, et al. Performance of Fully Automated Plasma Assays as Screening Tests for Alzheimer Disease-Related beta-Amyloid Status. JAMA Neurol. 2019.

8. Perez-Grijalba V, Arbizu J, Romero J, Prieto E, Pesini P, Sarasa L, et al. Plasma Abeta42/40 ratio alone or combined with FDG-PET can accurately predict amyloid-PET positivity: a cross-sectional analysis from the AB255 Study. Alzheimers Res Ther. 2019;11(1):96.

9. Vergallo A, Megret L, Lista S, Cavedo E, Zetterberg H, Blennow K, et al. Plasma amyloid beta 40/42 ratio predicts cerebral amyloidosis in cognitively normal individuals at risk for Alzheimer's disease. Alzheimers Dement. 2019;15(6):764-75.

10. Nakamura A, Kaneko N, Villemagne VL, Kato T, Doecke J, Dore V, et al. High performance plasma amyloid-beta biomarkers for Alzheimer's disease. Nature. 2018;554(7691):249-54.
